# COVID-19 vaccine hesitancy and attitudes toward routine vaccinations among Venezuelan migrants in Trinidad and Tobago: implications for a National Immunization Policy

**DOI:** 10.3389/fpubh.2024.1465762

**Published:** 2024-11-27

**Authors:** Nyla Lyons, Brendon Bhagwandeen, Blair Gopeechan, Jeffrey Edwards

**Affiliations:** ^1^Medical Research Foundation of Trinidad and Tobago, Port-of-Spain, Trinidad and Tobago; ^2^School of Mathematical and Computer Sciences, Heriot Watt University Malaysia, Putrajaya, Malaysia; ^3^Living Waters Community, Port-of-Spain, Trinidad and Tobago

**Keywords:** COVID-19, immunization, migrants, policy, Trinidad and Tobago, vaccine hesitancy, Venezuelan

## Abstract

**Introduction:**

Vaccine hesitancy poses a threat to the prevention of COVID-19 and other vaccine-controlled diseases. In 2019, the Government of Trinidad and Tobago launched a policy outlining the scope of health services in the public sector available to registered Venezuelan migrants to include access to routine immunizations. Little is understood about immunization uptake among migrants, including the uptake of COVID-19 vaccinations in Trinidad and Tobago.

**Method:**

Between July and October 2022, a survey was conducted using a sample of *n* = 507 Venezuelan migrants. We examined the relationship between COVID-19 vaccine hesitancy, migrant’s attitudes toward past vaccinations, their beliefs and perceptions about COVID-19 disease, and health-service related factors. Descriptive statistics summarized the characteristics of these migrants. Odds ratios with 95% confidence intervals and multivariable logistic regression was used to examine factors and attitudes associated with COVID-19 vaccine hesitancy.

**Results:**

Our findings showed that 89% of the migrants accessed publicly available health services while in Trinidad and Tobago, 72.4% reported that they did not refuse other vaccines in the past, and 23% reported being hesitant to take the COVID-19 vaccine. Females had higher odds of being COVID-19 vaccine hesitant compared to males, and participants expressing doubts about the source of vaccine information also had greater odds of vaccine hesitancy. Long waiting times at a public health clinic and costs associated with traveling to a clinic were associated with higher odds of COVID-19 vaccine hesitancy.

**Conclusion:**

A National Immunization Policy inclusive of the unmet needs of vulnerable migrant populations is needed to ensure equitable access to vaccinations.

## 1 Introduction

Trinidad and Tobago are a twin island republic at the southernmost point of the Caribbean with a population of approximately 1.36 million persons (1). Within the second half of the 20th century Trinidad and Tobago emerged as a prosperous, multicultural country having benefited significantly from its sizable oil and gas deposits (2). Due to its robust economy and geographical location, the country experienced a notable amount of intra-Caribbean and transnational migration spanning into the 21st century (3, 4). However, in the last decade it has experienced a new wave of mixed migration that now includes asylum seekers, refugees, victims of trafficking and other persons in need of international protection (3).

In the early half of 2017, the migrant and refugee population in Trinidad and Tobago consisted mostly of arrivals from Cuba, Syria, Jamaica, and Bangladesh. This refugee inflow intensified within the second half of 2017, primarily due to the humanitarian crisis in Venezuela - situated off the southwestern end of Trinidad and Tobago (4). According to an Interagency Participatory Assessment Report, as of 2022 approximately 34,100 Venezuelan refugees and vulnerable migrants were present in Trinidad and Tobago with over 22,000 officially registered as asylum seekers with the United Nations High Commissioner for Refugees (UNHCR) (5). In November 2022, the Government of Trinidad and Tobago acceded to the 1951 Convention relating to the Status of Refugees and its 1967 Protocol (Refugee Convention) (4). However, in the absence of a comprehensive refugee policy, the Government adopted, “A phased approach towards the establishment of a National Policy to address refugee and asylum matters in the Republic of Trinidad and Tobago” (6, 7). The current process for refugee status determination is conducted by the Office of the UNHCR, though plans of establishing and implementing national mechanisms to address refugee and asylum matters have been in development for several years.

Between mid-2020 and 2021, several cross-sectional studies conducted in the general population of Trinidad and Tobago revealed varying levels of COVID-19 vaccine hesitancy (8–10). A mid-2020 study by the Johns Hopkins Centre for Communication Programs found that 59% of respondents were hesitant to accept the COVID-19 vaccine (8). This early research captured the public’s apprehension at a time when misinformation, mistrust and concerns about safety were prevalent. Following the public availability of vaccines in April 2021, a Market Facts and Opinions (MFO) survey of 973 participants revealed that 65% were unwilling to accept the vaccine (9). This face-to-face, cross-sectional survey reflected growing concerns about safety and efficacy as the vaccine rollout began. By November 2021, a UNICEF-supported survey across several Caribbean countries, including Trinidad and Tobago, showed that of the 1,480 participants surveyed in the country, 35% were still hesitant (10). Safety and efficacy remained significant factors contributing to this reluctance. The variation in hesitancy rates across these studies can be attributed to the timing, as public perceptions of the vaccine evolved, and trust in health communication sources improved with the implementation of the National COVID-19 Vaccination Program and targeted communication aimed increasing confidence in the efficacy of the COVID-19 vaccine.

Vaccine hesitancy, particularly among vulnerable populations, remains a significant public health concern. While much of the research has focused on the general population, less has been published on the uptake of COVID-19 vaccines among vulnerable subgroups such as migrants and persons living with HIV in Trinidad and Tobago. These subgroups often face specific barriers contributing to hesitancy. One cross-sectional study examining COVID-19 vaccine hesitancy among persons living with HIV found that 39% of the 272 participants were hesitant, with those confident in the vaccine and its perceived benefits being less likely to be hesitant (11). Furthermore, a 2022 report by the International Organization for Migration (IOM) showed that 77% of 1,376 Venezuelans surveyed in Trinidad and Tobago were fully vaccinated (12), however the factors contributing to vaccine uptake and hesitancy among this subgroup of migrants and refugees are not clearly understood.

Our study explored the factors influencing COVID-19 vaccine hesitancy and attitudes toward routine vaccine uptake among Venezuelan migrants in Trinidad and Tobago. We also discuss the implications for a National Vaccination Policy and Programs that are inclusive of migrants and refugees, regardless of their legal status.

## 2 Materials and methods

### 2.1 Study design and participants

This cross-sectional study was conducted from June to October 2022 among Venezuelan migrants using telephone interviews and a structured questionnaire. The interviews were carried out by trained bilingual nurses. The study was conducted in accordance with the guidelines of the Helsinki Declaration and complying with the Trinidad and Tobago law on Medical Research involving human subjects. Approval from the Ministry of Health in Trinidad and Tobago was obtained for this study. The participants were recruited using simple random sampling from a database collated by the Archdiocese Ministry for Migrants & Refugees (AMMR). The AMMR identified the hidden migrant population through community networks, support organizations, outreach activities and religious institutions.

The exact size of the migrant sub population was largely unknown in Trinidad and Tobago. However, based on estimates by the AMMR, an approximated migrant population of 19,000 was used to determine a minimum sample size for the study. The sample size was calculated using a margin of error of 5%, power of 80%, a confidence level of 95% and a conservative estimated rate of vaccine hesitancy among migrants of 50%, giving a minimum sample size of *n* = 377 participants. This number was inflated by providing an additional allowance of 25% in sample recruitment due to possible non-response rate. A sample of *n* = 508 migrants was reached. Employing the use of bilingual nurses to overcome language, trust, and literacy barriers to participation contributed to the uptake of responses by the target population. The selected migrants were initially contacted to be sensitized about the survey with the nurses indicating that they would receive a follow-up call within one week to administer the survey. One record was discarded since this individual did not provide any responses, giving a final sample of *n* = 507 participants, and a response rate of 99.8%.

### 2.2 Measurements

The study questionnaire contained sociodemographic information, health status, use of health services, general vaccination behavior, the COVID-19 Vaccine Hesitancy Scale, and other potential factors of vaccine hesitancy. A pilot study was conducted to validate the questionnaire. After administering the questionnaire to *n* = 10 randomly selected migrants from the database, public health research experts reviewed the questionnaire design, content, words, comprehension, and ease of completion for face and content validity. Analysis of the components presented was believed to be important and relevant in the context of the study and the COVID-19 pandemic.

#### 2.2.1 Sociodemographic information

Sociodemographic variables included age, gender, residency in Trinidad and Tobago, education level and employment status.

#### 2.2.2 Health status, access to medical services, access to COVID-19 vaccine information and general vaccination behavior

The questionnaire captured medical conditions participants were previously diagnosed with, their access to medical services in Trinidad and Tobago, access to COVID-19 vaccine information, and used three questions to investigate participants’ attitudes toward vaccination:

1. Do you agree that vaccines can protect you from diseases?
2. Have you ever hesitated to get vaccination?
3. Have you ever refused to get vaccination?

#### 2.2.3 The COVD-19 Vaccine Hesitancy Scale

The COVID-19 Vaccine Hesitancy Scale was measured by 15 items based on previous studies (13, 14). Responses were collapsed into 3 categories: hesitant responses (2 points), unsure (1 point), and non-hesitant responses (0 points). The raw total score was calculated by summing each item’s score, ranging from 0–30. A linear transformation was used to convert the raw score to a 0–100 scale for which a score greater than or equal to 50 was COVID-19 vaccine hesitant (14). Sensitivity analysis was conducted and showed no differences in conclusions when using the continuous and dichotomous vaccine hesitancy outcome variables. Therefore, the dichotomous outcome variable was used in the final analysis for this study to facilitate comparison of COVID-19 vaccine hesitancy with other studies and meaningful interpretations in terms of odds ratios. The Cronbach’s *α* of the COVID-19 Vaccine Hesitancy Scale was *α* = 0.615 in this study, which was considered acceptable (15, 16). Table 1 provides the items present in the COVID-19 Vaccine Hesitancy Scale.

**Table 1:** COVID-19 Vaccine Hesitancy Scale items.

Items
Delay getting the COVID-19 vaccine for reasons other than illness/allergy. Decide not to get the COVID-19 vaccine for reasons other than illness/allergy. I want to get the COVID-19 vaccine recommended by the Government of Trinidad and Tobago. It is not necessary to get the COVID-19 vaccine at this time. The COVID-19 vaccine can prevent the COVID-19 virus. It is better to develop immunity by getting COVID-19. It is better to get fewer vaccines at the same time. I am concerned of the side effects from getting the COVID-19 vaccine. The COVID-19 vaccine might not be safe. I am concerned about the time taken to develop the vaccine. Recommend the COVID-19 vaccine to family and friends. Confident in the COVID-19 vaccine. Trust the information received about the COVID-19 vaccine. Openly discuss the COVID-19 vaccine with my doctor/other medical professionals. Trust a doctor’s advice to get the COVID-19 vaccine.

#### 2.2.4 Potential factors of COVID-19 vaccine hesitancy

These items were used to explore some of the potential barriers to getting the COVID-19 vaccine and factors related to COVID-19 vaccine hesitancy:

1. Individual attitudes toward COVID-19:

- Do you agree that the COVID-19 epidemic is a severe problem affecting the health of the community?
- Do you agree that COVID-19 will be a great threat to your health if you are infected?

1. Significant people’s advice:

- Do you agree that you will get the COVID-19 vaccine if doctors recommend it?
- Do you agree that the advice of your family members/friends will affect your intention of getting the COVID-19 vaccine?

1. Information about COVID-19 vaccine:

- Do you need transparent information about the COVID-19 vaccine development, efficacy and safety?
- Do you have doubts about the source of information about the COVID-19 vaccine?
- Have you ever received negative information about getting the COVID-19 vaccine?
- Would you still get the COVID-19 vaccine after receiving negative information about it?

1. Cost or time to get the COVID-19 vaccine:

- Do you agree that the time costs in waiting for the vaccination/staying at the clinic will be a barrier for you to get the COVID-19 vaccine?
- Do you agree that the environment of the clinic will be a barrier for you to get the COVID-19 vaccine?
- Do you agree that the cost of going to the clinic will be a barrier for you to get the COVID-19 vaccine?

1. Personal conditions:

- Do you agree that you have no need of getting the COVID-19 vaccine because you are healthy?
- Have you gotten emergency COVID-19 vaccination for some reason?

### 2.3 Statistical analysis

Data were cleaned using Microsoft Excel, coded, and analyzed using Statistical Product and Service Solutions (SPSS) (IBM SPSS Statistics for Windows, Version 28.0, Armonk, NY). Participants were grouped into two categories: COVID-19 vaccine hesitant (1) or not COVID-19 vaccine hesitant (0), based on whether their scores on the transformed COVID-19 Vaccine Hesitancy Scale were greater than or equal to 50. Categorical variables in sociodemographic data, health status, use of health services, general vaccination behavior, access to COVID-19 information and potential factors of vaccine hesitancy were presented as frequencies and percentages. Ungrouped ages were presented as mean ± standard deviation (SD). Bivariate analyses examined the relationship between vaccine hesitancy and baseline characteristics. Explanatory binary logistic regression was conducted to examine associations between COVID-19 vaccine hesitancy with sociodemographic variables, general vaccine behavior and the potential factors considered, using unadjusted odds ratios (OR) with 95% confidence intervals (CI). *A priori* possible predictors of COVID-19 vaccine hesitancy with *p* < 0.05 in bivariate analyses were then included in a multivariable logistic regression model. Participants with “unsure”/“do not know” responses were excluded from the logistic regression analysis. The goodness-of-fit of the model was assessed by the Hosmer-Lemeshow test and the area under the curve (AUC) of the receiver operating characteristic (ROC) curve. Statistical significance for this study was set at *p* < 0.05.

### 2.4 Ethical approval

The study protocol, data collection instruments and informed consent form were approved by the Ministry of Health of Trinidad and Tobago. Formal consent was provided by each individual participating in this study.

## 3 Results

### 3.1 Characteristics of migrants

Table 2 summarizes the sociodemographic characteristics of participants. A total of *n* = 507 respondents were included in this study. The average age of participants was 33.83 ± 9.72 years. Overall, 438 (86.4%) participants were residing in Trinidad and Tobago for more than 24 months, 396 (78.1%) were female and 337 (66.5%) received post-secondary schooling. Participants largely resided in areas such as Arima and Environs (*n* = 79, 15.6%), the Borough of Chaguanas (*n* = 72, 14.2%) and Penal/Debe (*n* = 60, 11.8%). Overall, 311 (61.4%) respondents were employed (either part-time or full-time), 287 (56.6%) lost their jobs or had their income reduced due to the COVID-19 pandemic while living in Trinidad and Tobago, 455 (89.7%) did not have close family over 60 years of age, and 266 (52.5%) had between three to five persons residing in their households.

**Table 2:** Sociodemographic characteristics of participants.

Variables	
Age (years) (mean ± SD)	33.83 ± 9.72
	*n* (%)
Length of residency	
Less than 6 months	6 (1.2)
6–12 months	15 (3.0)
12–24 months	48 (9.5)
More than 24 months	438 (86.4)
Total	507 (100.0)
Gender	
Male	111 (21.9)
Female	396 (78.1)
Total	507 (100.0)
Education level	
Primary schooling	21 (4.1)
Secondary schooling	149 (29.4)
Post-secondary schooling	337 (66.5)
Total	507 (100.0)
Area of residence	
City of Port-of-Spain	45 (8.9)
Borough of Chaguanas	72 (14.2)
Arima and Environs	79 (15.6)
San Juan/Lavantille	11 (2.2)
Mayaro/Rio Claro	2 (0.4)
Penal/Debe	60 (11.8)
Borough of Point Fortin	3 (0.6)
City of San Fernando	43 (8.5)
Diego Martin	42 (8.5)
Tunapuna/Piarco	21 (4.1)
Sangre Grande	12 (2.4)
Siparia	10 (2.0)
Couva/Tabaquite/Talparo	18 (3.6)
Princess Town	13 (2.6)
Other^1^	76 (15.9)
Total	507 (100.0)
Employment status	
Unemployed	196 (38.7)
Employed part-time^2^	158 (31.2)
Employed full-time	153 (30.2)
Total	507 (100.0)
Lost job/had income reduction due to COVID-19 pandemic	
No	220 (43.4)
Yes, due to quarantine period	194 (38.3)
Yes, due to restriction measures	93 (18.3)
Total	507 (100.0)
Close family over 60 years old	
No	455 (89.7)
Yes, but not living together	32 (6.3)
Yes, and living together	20 (3.9)
Total	507 (100.0)
Number of persons at residence	
Two or less	69 (13.6)
Three to five	266 (52.5)
More than five	172 (33.9)
Total	507 (100.0)

^1Included areas such as Arouca, Bamboo, Barrackpore, Cedros, Chaguaramas, Claxton Bay, Cunupia, Curepe, El Socorro, Freeport, Fyzabad, Gasparillo, Piarco, Rancho Quemado, Santa Cruz, Santa Elena, Valencia and Valsayn. 2Included self-employed or otherwise.^

### 3.2 Health status, access to medical services, access to COVID-19 vaccine information, and general vaccination behavior

#### 3.2.1 Health status

**Figure 1:**
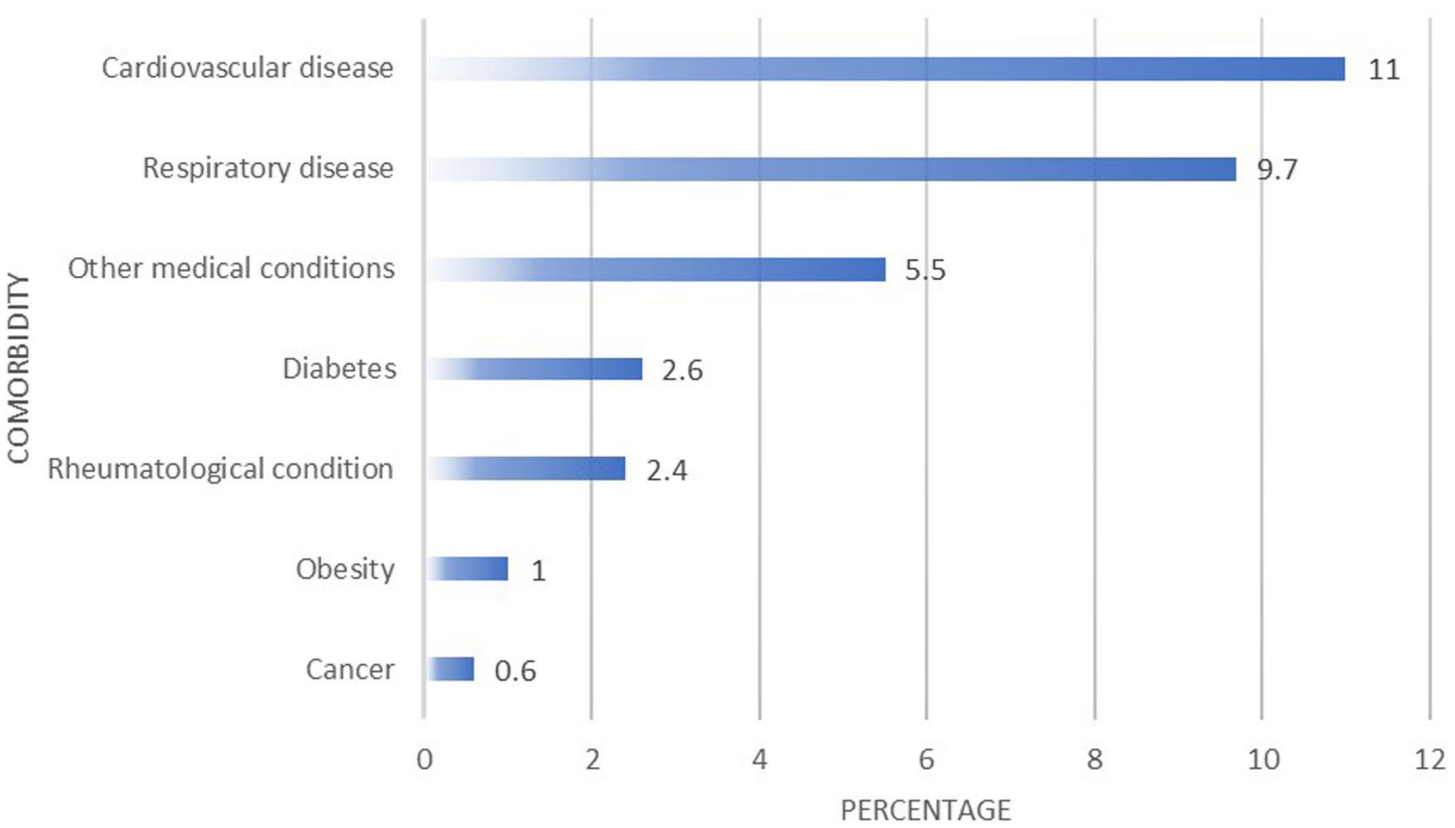
Health status of migrants.

In total, 56 (11%) participants reported being previously diagnosed with cardiovascular diseases (such as hypertension and heart disease) and 49 (9.7%) had a respiratory disease (such as asthma). Figure 1 presents other medical conditions reported by the migrants. Three (0.6%) migrants reported having multiple comorbidities.

#### 3.2.2 Access to medical services

**Figure 2:**
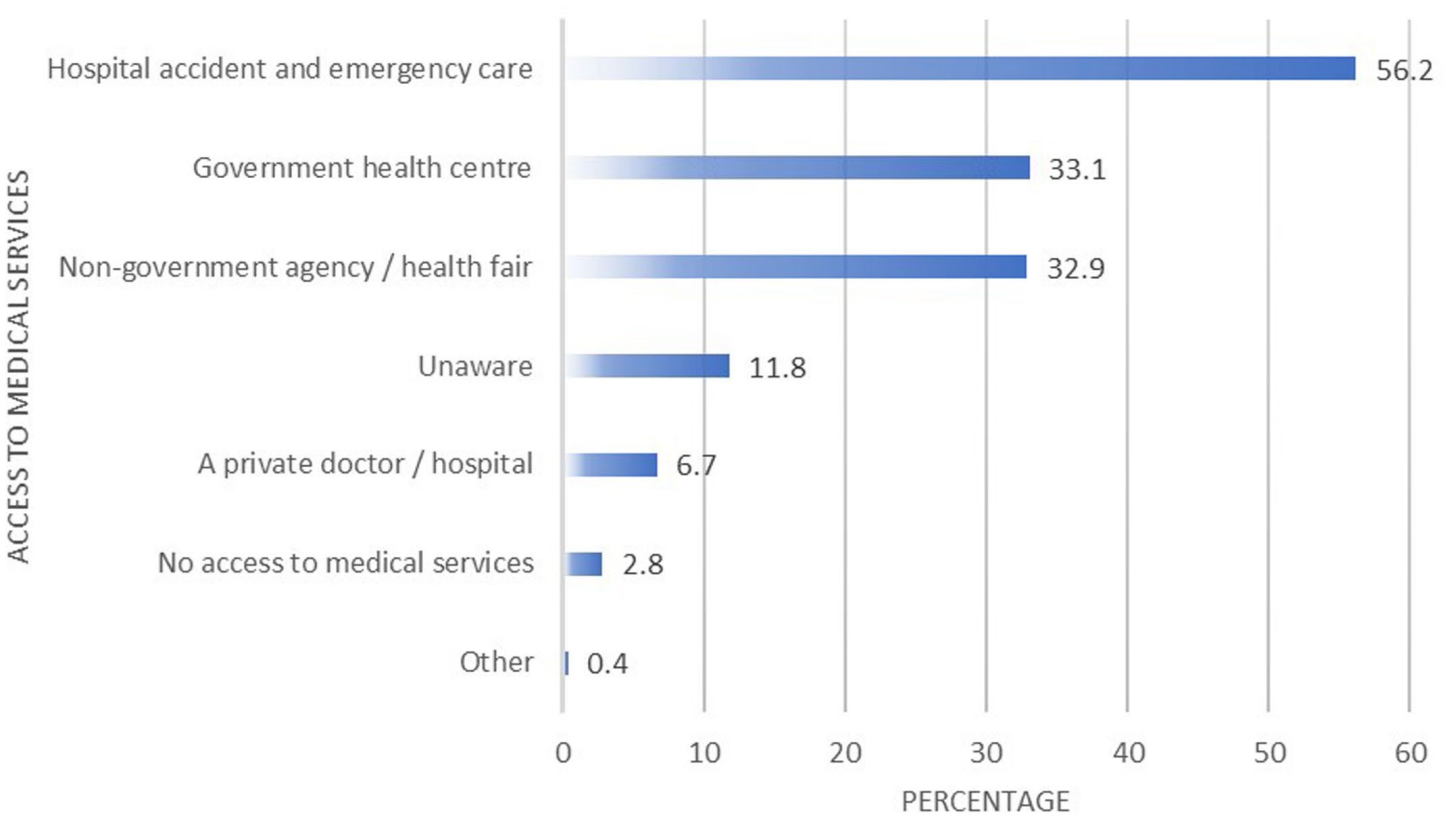
Migrants’ access to medical services.

Most of the participants (*n* = 285, 56.2%) accessed medical services through the accident and emergency care at hospitals. Figure 2 presents the avenues through which migrants accessed medical services while in Trinidad and Tobago.

#### 3.2.3 COVID-19 and general vaccination behavior

Most participants (*n* = 350, 69%) indicated that they had not previously contracted COVID-19. If offered the COVID-19 vaccine in the next 2 months, 157 (31%) participants indicated that they were unlikely to accept the vaccine.

Table 3 presents general vaccination behaviors of participants. Overall, 332 (65.5%) participants were unsure about whether vaccines could provide protection from diseases, 367 (72.4%) indicated that they never hesitated to get vaccinations in the past, and 384 (75.7%) did not previously refuse to get vaccinations.

**Table 3:** General vaccination behavior of participants.

Variable	*n* (%)
Do you agree that vaccines can protect you from diseases?	
No	52 (10.3)
Yes	123 (24.3)
Unsure	332 (65.5)
Total	507 (100.0)
Have you ever hesitated to get vaccination?	
No	367 (72.4)
Yes	52 (10.3)
Undisclosed	88 (17.4)
Total	507 (100.0)
Have you ever refused to get vaccination?	
No	384 (75.7)
Yes	47 (9.3)
Undisclosed	76 (15.0)
Total	507 (100.0)

#### 3.2.4 Access to COVID-19 information

**Figure 3:**
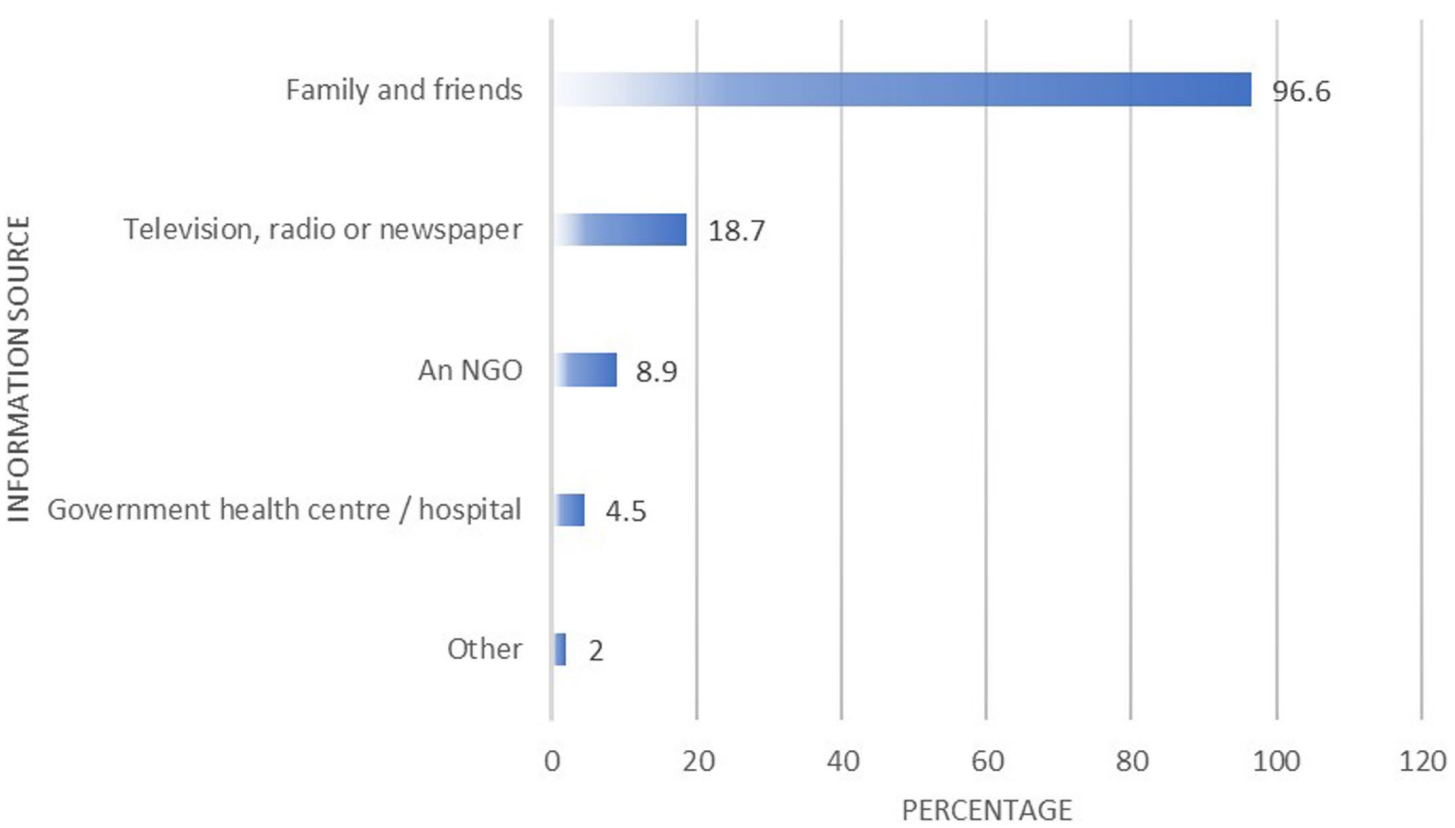
Migrants’ COVID-19 vaccine information source.

Most participants (*n* = 490, 96.6%) indicated that they received information on the COVID-19 vaccine from family and friends. Figure 3 presents migrants’ access to COVID-19 information.

### 3.3 Attitudes toward COVID-19 vaccine hesitancy

Table 4 summarizes potential attitudinal factors of COVID-19 vaccine hesitancy. In this study, 114 (22.5%) participants were observed as COVID-19 vaccine hesitant. Most participants agreed that the COVID-19 epidemic was a severe problem affecting the health of their community (*n* = 402, 79.3%) and that they would get the COVID-19 vaccine if doctors recommended it (*n* = 394, 77.7%). Most persons also previously received negative information about getting the COVID-19 vaccine (*n* = 363, 71.6%) but would still get the COVID-19 vaccine after receiving negative information about it (*n* = 371, 73.2%).

**Table 4:** COVID-19 vaccine hesitancy and attitudinal factors.

Variables	*n* (%)
COVID-19 vaccine hesitancy	
Not hesitant	393 (77.5)
Hesitant	114 (22.5)
Total	507 (100.0)
Do you agree that the COVID-19 epidemic is a severe problem affecting the health of the community?	
No	-
Yes	402 (79.3)
Unsure	105 (20.7)
Total	507 (100.0)
Do you agree that COVID-19 will be a great threat to your health if you are infected?	
No	4 (0.8)
Yes	338 (66.7)
Unsure	165 (32.5)
Total	507 (100.0)
Do you agree that you will get the COVID-19 vaccine if doctors recommend it?	
No	56 (11.0)
Yes	394 (77.7)
Unsure	57 (11.2)
Total	507 (100.0)
Do you agree that the advice of the family members/friends will affect your intention of getting the COVID-19 vaccine?	
No	303 (59.8)
Yes	158 (31.2)
Unsure	46 (9.0)
Total	507 (100.0)
Do you need information about the COVID-19 vaccine development, efficacy and safety?	
No	57 (11.2)
Yes	275 (54.2)
Unsure	175 (34.5)
Total	507 (100.0)
Do you have doubts about the source of information about the COVID-19 vaccine?	
No	88 (17.4)
Yes	170 (33.5)
Unsure	249 (49.1)
Total	507 (100.0)
Have you ever received negative information about getting the COVID-19 vaccine?	
No	134 (26.4)
Yes	363 (71.6)
Unsure	10 (2.0)
Total	507 (100.0)
Would you still get the COVID-19 vaccine after receiving negative information about it?	
No	100 (19.7)
Yes	371 (73.2)
Unsure	36 (7.1)
Total	507 (100.0)
Do you agree that the time spent waiting for the vaccination/staying at the clinic will be a barrier for you to get the COVID-19 vaccine?	
No	391 (77.1)
Yes	22 (4.3)
Unsure	94 (18.5)
Total	507 (100.0)
Do you agree that the environment of the clinic will be a barrier for you to get the COVID-19 vaccine?	
No	381 (75.1)
Yes	29 (5.7)
Unsure	97 (19.1)
Total	507 (100.0)
Do you agree that the cost of going to the clinic will be a barrier for you to get the COVID-19 vaccine?	
No	291 (57.4)
Yes	109 (21.5)
Unsure	107 (21.1)
Total	507 (100.0)
Do you agree that you have no need to get the COVID-19 vaccine because you are healthy?	
No	187 (36.9)
Yes	14 (2.8)
Unsure	306 (60.4)
Total	507 (100.0)
Have you gotten emergency COVID-19 vaccination for some reason?	
No	419 (82.6)
Yes	6 (1.2)
Unsure	82 (16.2)
Total	507 (100.0)

### 3.4 Factors associated with COVID-19 vaccine hesitancy among Venezuelan migrants in Trinidad and Tobago

#### 3.4.1 Associations between COVID-19 vaccine hesitancy and sociodemographic characteristics

Table 5 presents bivariate associations between COVID-19 vaccine hesitancy and sociodemographic characteristics. Gender was significantly associated with COVID-19 vaccine hesitancy (*p* < 0.05). There were greater odds of COVID-19 vaccine hesitancy in females (OR 1.794, 95% CI 1.019–3.156) relative to males.

**Table 5:** Bivariate associations and unadjusted odds ratios (with 95% CI) of COVID-19 vaccine hesitancy and sociodemographic characteristics.

Variables	COVID-19 vaccine hesitancy (Ref: Not hesitant)	
	Unadjusted OR (95% CI)	*p*-value
Age (years) (mean ± SD)	0.978 (0.956–1.001)	0.060
Length of residency		
Less than 6 months*	1.000	
6–12 months	0.308 (0.032–2.942)	0.306
12–24 months	0.743 (0.121–4.552)	0.748
More than 24 months	0.569 (0.103–3.153)	0.519
Gender		
Male*	1.000	
Female	1.794 (1.019–3.156)	0.043
Education level		
Primary schooling*	1.000	
Secondary schooling	0.483 (0.179–1.306)	0.152
Post-secondary schooling	0.602 (0.235–1.545)	0.291
Area of residence		
City of Port-of-Spain*	1.000	
Borough of Chaguanas	0.238 (0.087–0.650)	0.005
Arima and Environs	0.751 (0.334–1.687)	0.487
San Juan/Lavantille	0.830 (0.191–3.609)	0.804
Mayaro/Rio Claro	2.214 (0.129–38.004)	0.584
Penal/Debe	0.443 (0.175–1.119)	0.085
Borough of Point Fortin	-	-
City of San Fernando	0.506 (0.187–1.368)	0.179
Diego Martin	0.993 (0.400–2.463)	0.987
Tunapuna/Piarco	0.692 (0.211–2.266)	0.543
Sangre Grande	2.214 (0.606–8.090)	0.229
Siparia	0.554 (0.104–2.950)	0.488
Couva/Tabaquite/Talparo	0.277 (0.056–1.371)	0.116
Princess Town	0.185 (0.022–1.561)	0.121
Other	0.902 (0.404–2.013)	0.801
Employment status		
Unemployed*	1.000	
Employed part-time	0.676 (0.408–1.120)	0.128
Employed full-time	0.704 (0.424–1.167)	0.173
Lost job/had income reduction due to COVID-19 pandemic		
No*	1.000	
Yes, due to quarantine period	0.707 (0.442–1.132)	0.149
Yes, due to restriction measures	0.930 (0.527–1.639)	0.801
Close family over 60 years old		
No*	1.000	
Yes, but not living together	1.635 (0.749–3.566)	0.217
Yes, and living together	1.199 (0.425–3.379)	0.732
Number of persons at residence		
Two or less*	1.000	
Three to five	1.175 (0.601–2.298)	0.638
More than five	1.481 (0.740–2.964)	0.267

*Reference category.

There were no significant associations between participants’ ages, length of time residing in Trinidad and Tobago, highest level of education attained, area of residence in Trinidad and Tobago, current employment status, having lost their job or had an income reduction due to the COVID-19 pandemic, having close family members of 60 years of age and the number of persons living in the household and COVID-19 vaccine hesitancy (*p* > 0.05).

#### 3.4.2 Associations between COVID-19 vaccine hesitancy, health status, access to medical services, access to COVID-19 vaccine information and general vaccination behavior

Table 6 presents bivariate associations between COVID-19 vaccine hesitancy, health status, access to medical services, access to COVID-19 vaccine information and general vaccination behaviors. General vaccination behaviors were significantly associated with COVID-19 vaccine hesitancy (*p* < 0.05). Persons who agreed (OR 0.317, 95% CI 0.140–0.718) that vaccines could protect them from diseases had lower odds of being COVID-19 vaccine hesitant relative to persons who did not agree.

**Table 6:** Bivariate associations and unadjusted odds ratios (with 95% CI) of COVID-19 vaccine hesitancy according to health status, access to medical services, access to COVID-19 vaccine information and general vaccine attitudes.

Variables	COVID-19 vaccine hesitancy (Ref: Not hesitant)	
	Unadjusted OR (95% CI)	*p*-value
Comorbidity present		
No*	1.000	
Yes	1.067 (0.683–1.668)	0.775
Access to medical services		
No*	1.000	
Yes	0.511 (0.168–1.556)	0.237
Previously contracted COVID-19		
No*	1.000	
Suspected symptoms but not tested	0.460 (0.157–1.348)	0.157
Yes, with no symptoms	0.402 (0.050–3.262)	0.394
Yes, with mild symptoms	0.969 (0.582–1.614)	0.904
Yes, with severe symptoms	0.460 (0.056–3.789)	0.470
Do you agree that vaccines can protect you from diseases?		
No*	1.000	
Yes	0.317 (0.140–0.718)	0.006
Have you ever hesitated to get vaccination?		
No*	1.000	
Yes	12.381 (6.499–23.587)	< 0.001
Have you ever refused to get vaccination?		
No*	1.000	
Yes	15.296 (7.711–30.346)	< 0.001
Self-reported COVID-19 vaccination likelihood		
Very unlikely*	1.000	
Somewhat unlikely	1.087 (0.466–2.535)	0.846
Neutral	1.749 (0.943–3.245)	0.076
Somewhat likely	0.842 (0.418–1.697)	0.630
Very likely	1.557 (0.817–2.969)	0.178

*Reference category.

Persons who previously hesitated to get vaccinations (OR 12.381, 95% CI 6.499–23.587) had increased odds of COVID-19 vaccine hesitancy relative to persons did not hesitate for previous vaccinations. Persons who previously refused to get vaccinations (OR 15.296, 95% CI 7.711–30.346) also had greater odds to be COVID-19 vaccine hesitant relative to persons did not refuse previous vaccinations.

Presence of comorbidities, having access to medical services, having previously contracted COVID-19 vaccine and participants’ self-reported likelihood of COVID-19 vaccination if offered the vaccine within the next 2 months were not significantly associated with COVID-19 vaccine hesitancy (*p* > 0.05).

#### 3.4.3 Associations between COVID-19 vaccine hesitancy and attitudinal factors

Table 7 presents bivariate associations between COVID-19 vaccine hesitancy and attitudinal factors. Other attitudinal factors were significantly associated with COVID-19 vaccine hesitancy (*p* < 0.05). Persons who agreed (OR 0.075, 95% CI 0.040–0.141) that they would get the COVID-19 vaccine if doctors recommended it had lower odds to be COVID-19 vaccine hesitant when compared with persons who did not agree. Persons who agreed (OR 2.977, 95% CI 1.886–4.699) that the advice of family members or friends would affect their intentions of getting the COVID -19 vaccine had increased odds of being COVID-19 vaccine hesitant when compared with persons did not believe they would be influenced.

**Table 7:** Bivariate associations and unadjusted odds ratios (with 95% CI) of COVID-19 vaccine hesitancy and attitudinal factors.

Variables	COVID-19 vaccine hesitancy (Ref: Not hesitant)	
	Unadjusted OR (95% CI)	*p*-value
Do you agree that COVID-19 will be a great threat to your health if you are infected?		
No*	1.000	
Yes	0.097 (0.010–0.943)	0.044
Do you agree that you will get the COVID-19 vaccine if doctors recommend it?		
No*	1.000	
Yes	0.075 (0.040–0.141)	< 0.001
Do you agree that the advice of family members/friends will affect your intention of getting the COVID-19 vaccine?		
No*	1.000	
Yes	2.977 (1.886–4.699)	< 0.001
Do you need information about COVID-19 vaccine development, efficacy, and safety?		
No*	1.000	
Yes	0.421 (0.226–0.785)	0.007
Do you have doubts about the source of information about the COVID-19 vaccine?		
No*	1.000	
Yes	5.039 (2.364–10.742)	< 0.001
Did not ever receive negative information about getting the COVID-19 vaccine?		
No*	1.000	
Yes	0.816 (0.509–1.309)	0.399
Would you still get the COVID-19 vaccine after receiving negative information about it?		
No*	1.000	
Yes	0.104 (0.063–0.174)	< 0.001
Do you agree that the time spent waiting for the vaccination/staying at the clinic will be a barrier for you to get the COVID-19 vaccine?		
No*	1.000	
Yes	5.816 (2.386–14.178)	< 0.001
Do you agree that the environment of the clinic will be a barrier for you to get the COVID-19 vaccine?		
No*	1.000	
Yes	2.979 (1.285–6.908)	0.011
Do you agree that the cost of going to the clinic will be a barrier for you to get the COVID-19 vaccine?		
No*	1.000	
Yes	1.173 (0.642–2.141)	0.604
Do you agree that you have no need to get the COVID-19 vaccine because you are healthy?		
No*	1.000	
Yes	40.750 (8.588–193.355)	< 0.001
Have you gotten emergency COVID-19 vaccination for some reason?		
No*	1.000	
Yes	0.763 (0.088–6.618)	0.806

*Reference category.

Persons who needed more information (OR 0.421 95% CI 0.226–0.785) about COVID-19 vaccine development, efficacy and safety had lower odds to be COVID-19 vaccine hesitant than persons who did not need information about the vaccine. Persons having doubts (OR 5.039, 95% CI 2.364–10.742) about the source of information about the COVID-19 vaccine had greater odds of COVID-19 vaccine hesitancy when compared with persons without any doubts. Persons who would still get the COVID-19 vaccine (OR 0.104, 95% CI 0.063–0.174) after receiving negative information about it were less likely to be COVID-19 vaccine hesitant compared with persons did not intend to still get the vaccine.

Persons believing (OR 5.816, 95% CI 2.386–14.178) that the time spent waiting for the vaccination or staying at the clinic would be a barrier to get the COVID-19 vaccine had greater odds to be COVID-19 vaccine hesitant than those who did not believe the waiting time to be a barrier. Persons believing (OR 2.979, 95% CI 1.285–6.908) that the environment of the clinic would be a barrier to get the COVID-19 vaccine also had increased odds to be COVID-19 vaccine hesitant than those who did not believe the environment to be a barrier. Persons believing (OR 40.750, 95% CI 8.588–193.355) that there was no need to get the COVID-19 vaccine because they were healthy had increased odds of being COVID-19 vaccine hesitant than those who did not have this belief.

There were no significant associations between beliefs that the COVID-19 epidemic was a severe problem affecting the health of the community, beliefs that COVID-19 would be a great threat to their health if infected, previously having emergency COVID-19 vaccination and COVID-19 vaccine hesitancy (*p* > 0.05).

### 3.5 Predictors of COVID-19 vaccine hesitancy

**Figure 4:**
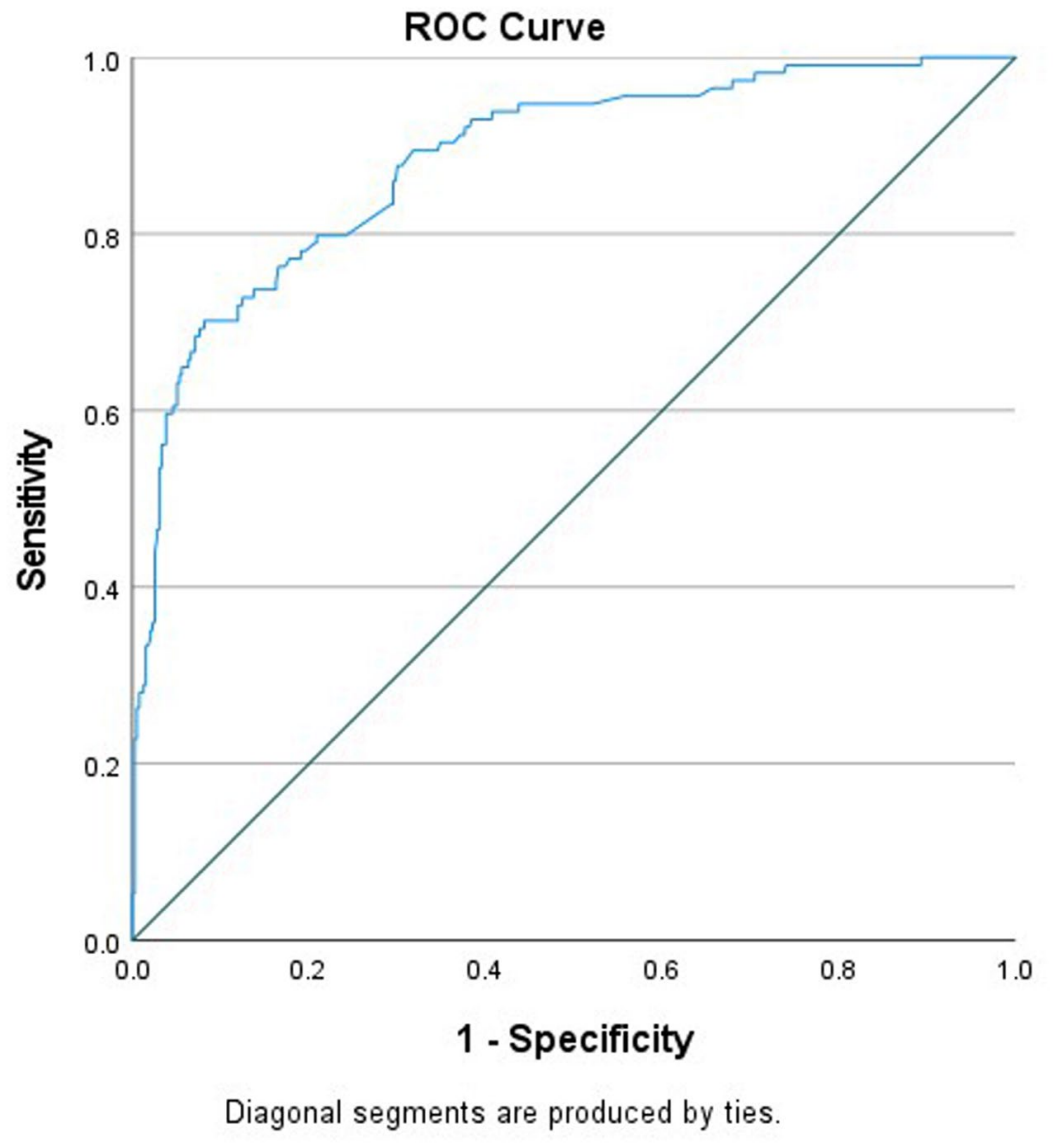
ROC curve for migrant COVID-19 vaccine hesitancy model.

Table 8 presents the results of the multivariable logistic regression model to determine predictors of COVID-19 vaccine hesitancy among participants. Significant factors from the bivariate analyses (*p* < 0.05) were used to assess these potential predictors (*n* = 175 participants were used in the multivariable analysis after excluding “unsure”/“do not know” responses, which still met the minimum requirements of 10 observations per variable in a multivariable model (17, 18)). The Hosmer and Lemeshow test revealed that the model was a good fit (*p* > 0.05). The area under the receiver operating characteristic (ROC) curve was 0.884 (*p* < 0.05, 95% CI 0.848–0.920), which indicated that the model displayed good discrimination ability between hesitant and non-hesitant persons (Figure 4).

**Table 8:** Adjusted odds ratios (with 95% CI) of COVID-19 vaccine hesitancy with sociodemographic, health-related and vaccination behaviors and COVID-19 attitudinal characteristics.

Variables	COVID-19 vaccine hesitancy (Ref: Not hesitancy)		*p*-value
	Adjusted OR	95% CI	
Gender			
Male*	1.000		
Female	3.648	1.563–8.514	0.003
Do you agree that vaccines can protect you from diseases?			
No*	1.000		
Yes	0.106	0.024–0.471	0.003
Have you ever hesitated to get vaccination?			
No*	1.000		
Yes	0.953	0.195–4.649	0.953
Have you ever refused to get vaccination?			
No*	1.000		
Yes	4.492	0.937–21.543	0.060
Do you agree that you will get the COVID-19 vaccine if doctors recommend it?			
No*	1.000		
Yes	0.269	0.096–0.755	0.013
Do you agree that the advice of family members/friends will affect your intention of getting the COVID-19 vaccine?			
No*	1.000		
Yes	1.181	0.563–2.476	0.660
Do you need information about COVID-19 vaccine development, efficacy and safety?			
No*	1.000		
Yes	1.840	0.646–5.246	0.254
Do you have doubts about the source of information about the COVID-19 vaccine?			
No*	1.000		
Yes	1.798	0.593–5.452	0.300
Did not ever receive negative information about getting the COVID-19 vaccine?			
No*	1.000		
Yes	0.406	0.205–0.807	0.010
Would you get the COVID-19 vaccine after receiving negative information about it?			
No*	1.000		
Yes	0.362	0.126–1.040	0.059
Do you agree that the time spent waiting for the vaccination/staying at the clinic will be a barrier for you to get the COVID-19 vaccine?			
No*	1.000		
Yes	2.704	0.707–10.341	0.146
Do you agree that the environment of the clinic will be a barrier for you to get the COVID-19 vaccine?			
No*	1.000		
Yes	1.614	0.388–6.713	0.510
Do you agree that the cost of going to the clinic will be a barrier for you to get the COVID-19 vaccine?			
No*	1.000		
Yes	1.798	0.680–4.756	0.237
Do you agree that you have no need to get the COVID-19 vaccine because you are healthy?			
No*	1.000		
Yes	16.362	1.821–146.999	0.013

*Reference category.

In the multivariable model, while controlling for attitudinal factors, females remained with greater odds to be COVID-19 vaccine hesitant (AOR 3.648, 95% CI 1.563–8.514) relative to males. Persons who believed that vaccines could protect them from diseases remained with lower odds of being COVID-19 vaccine hesitant (AOR 0.106, 95% CI 0.024–0.471) relative to those who did not have this perception, while persons who would get the COVID-19 vaccine if doctors recommended it also displayed lower odds of being COVID-19 vaccine hesitant (AOR 0.269, 95% CI 0.096–0.755) relative to persons who would not listen to the doctors’ advice.

Persons who did not receive negative information about getting the COVID-19 vaccine had lower odds of COVID-19 vaccine hesitancy (AOR 0.406, 95% CI 0.205–0.807) relative to persons who received negative information about the vaccine, and persons who believed there was no need to get the COVID-19 vaccine because they were healthy (AOR 16.3621.821–146.999) had greater odds of being COVID-19 vaccine hesitant relative to persons who did not have this belief.

## 4 Discussion

Our study examined the determinants of COVID-19 vaccine hesitancy among migrants, attitudes toward past vaccinations, their beliefs and perceptions about COVID-19 and health service factors. We also discussed the implications for national immunization policies and programs. This research was conducted between July and September 2022, one year after the rollout of the National COVID-19 Vaccination Program in Trinidad and Tobago.

Our results showed that 89% of participants reported accessing publicly available health services while living in Trinidad and Tobago. The high proportion of migrants reporting access to publicly available health services can be attributed to the government’s efforts to formalize the status of Venezuelan refugees and migrants in the country. Beginning in April 2019, in response to the increasing influx of Venezuelan refugees, the government initiated a two-week registration exercise which legalized approximately 16,000 Venezuelans, granting them access to primary care and emergency health services, including immunizations. This effort significantly improved healthcare access for those registered. A second registration exercise in 2021, which offered extensions to previously registered individuals, excluded new arrivals, thereby limiting healthcare access for many, particularly during the COVID-19 pandemic.

At the time of our study, 23% of migrants reported hesitancy toward taking the COVID-19 vaccine. The National COVID-19 Vaccination Program was rolled out by the government in April 2021, prioritizing individuals aged 60 years and older, those with non-communicable diseases (such as hypertension, diabetes, cancer, heart, and respiratory diseases), and healthcare workers. The Vaccination Program was supported by public education campaigns designed to increase awareness of the vaccine’s safety and efficacy, correct misinformation, and promote widespread uptake across all socioeconomic groups, particularly among those disproportionately affected by disease.

By July 2021, in collaboration with civil society refugee organizations, the government expanded its efforts, aiming to improve access to community-based testing and increase COVID-19 vaccine uptake for refugees and migrants. No data is available on the number of refugees and migrants reached because of these efforts. A 2022 report by the International Organization for Migration (IOM) revealed that 77% of 1,376 Venezuelans surveyed were fully vaccinated. The data, collected between April and June 2021, highlighted significant progress in vaccinating Venezuelan migrants but did not account for the 23% who remained unvaccinated at that time. Therefore, our study was the first national undertaking to explore vaccine uptake, hesitancy and associated factors among Venezuelan migrants and refugees in Trinidad and Tobago. Our study found significant associations between COVID-19 vaccine hesitancy and gender, beliefs about vaccine efficacy, and risk perceptions regarding COVID-19.

Throughout the COVID-19 pandemic, studies conducted among refugees and migrants in various settings reported between 18 to 30% vaccine hesitancy in the respective populations (19–23). Early in the pandemic response, a large cross-sectional survey conducted in a migrant-majority population in Qatar reported that 20.2% of the 6,882 migrants surveyed were vaccine hesitant (19). Migrants in this study reported concerns about the safety of the vaccine (53.8%), its long-term side effects (47.9%) and a lack of confidence in the vaccine (43.4%) as the main contributing factors. In the United States, a cross-sectional study using snowball sampling was conducted to assess acceptance of the COVID-19 vaccine among refugees (22). The results of the study reported that among the 435 respondents, 7.6% did not plan to receive a vaccine, and 22.1% were unsure. Factors contributing to hesitancy included the perceived potential side effects (71.3%) and concerns regarding vaccine effectiveness (12.4%). A later study conducted among migrant populations residing in Germany, administered after vaccines were initially available, reported that 18.9% of migrants did not agree that the vaccine was important, with 204 survey participants reported having received at least one dose of the COVID-19 vaccine (20). Migrants in the study also reported the fear of the vaccine side effects (55%), and 47.9% viewed the vaccine as unsafe. In this study of vaccine hesitancy among migrant populations, 31.3% of participants reported having more confidence in taking that vaccine if it was endorsed by their doctor compared to 9.2% percent who were vaccine hesitant.

Females in our study had higher odds of being vaccine hesitant than males, which is consistent with research that links gender, perceptions of disease risks, and vaccine hesitancy among immigrant populations. For instance, an online cross-sectional survey conducted among 388 first and second generation African and Caribbean immigrants in the United States, found that women were more likely to be vaccine hesitant compared to men (24). Furthermore, another online survey completed by 498 foreign migrants in China reported that female migrants were less likely to believe in the efficacy of the COVID-19 vaccine (25). Studies also underscored other significant sociodemographic factors such as age, socioeconomic factors and education contributing to vaccine uptake and hesitancy (19, 24, 25). Alabdulla et al. (19) noted that apart from being female, respondents of high socioeconomic status were more vaccine hesitant compared to their counterparts who were generally younger and living without families. Together, these findings underscore the complex role of gender in shaping vaccine behaviors, aligning with our study’s findings. These associations highlighted a critical need for strategies to reach vulnerable migrant women and increase their access to and uptake of COVID-19 vaccines to reduce their risk of transmission to families and offspring. In various settings, women play a vital role in the uptake of immunizations for their children, underscoring the need for immunization policies to be inclusive of the needs of pregnant migrant women and to ensure the availability of vaccination at public antenatal care clinics.

With regards to attitudes toward overall vaccination, our study showed 72% of migrants reported not refusing to get vaccinated in the past. While the question did not specify whether this pertained to while living in Trinidad and Tobago, it highlighted a generally positive attitude toward vaccination among the migrant population. However, the COVID-19 pandemic introduced new and unique challenges that likely influenced vaccine hesitancy. First, the speed of vaccine development, emergency use authorizations, and widespread misinformation and conspiracy theories circulating online may have raised concerns about the vaccine’s safety and efficacy. Migrants, often facing language barriers and limited access to reliable information, may have been more vulnerable to misinformation, particularly if outreach efforts were not adequately tailored to their communities. Second, the pandemic amplified existing socio-economic vulnerabilities. Migrants, especially those from marginalized communities, may have faced additional stress due to job loss, housing instability, and difficulty accessing healthcare, which could have exacerbated their mistrust in public health interventions. Furthermore, the exclusion of new arrivals from registration programs after 2021 limited healthcare access for some migrants, possibly heightening their reluctance to engage with the health system. In this context, the pandemic’s uncertainties, compounded by economic and social stressors, likely played a significant role in driving the 23% of COVID-19 vaccine hesitancy observed in our study.

Our study also explored access to health services and found that individuals who perceived barriers such as long waiting times, poor clinic environments, and high costs associated with clinic visits were more likely to be hesitant about the COVID-19 vaccine compared to those who did not view these factors as barriers. In the context of Trinidad and Tobago, this highlights the way migrants perceive barriers to accessing public healthcare. For those who did not benefit from the government’s 2019 Registration Exercise, fears of deportation may prevent them from seeking public health services, leading to limited healthcare access. Unregistered migrants often have no option but to seek care in private clinics, where the costs of services are perceived as prohibitive, exacerbating vaccine hesitancy.

This is consistent with studies showing that financial barriers, alongside difficulty understanding healthcare systems, language challenges, and low trust in the health system, as being common obstacles for migrants accessing health services (26–30). For example, research indicates that migrants often prefer free vaccination and are less likely to pay for COVID-19 vaccines, which further supports our findings regarding cost as a key barrier (31). Additionally, no significant associations were found in our study between health factors such as having a medical comorbidity, previously contracting COVID-19, or accessing medical services and COVID-19 vaccine hesitancy. Similarly, beliefs about the severity of the COVID-19 epidemic or the perceived personal health threat posed by COVID-19 did not significantly influence vaccine hesitancy. These findings emphasize the importance of addressing systemic barriers to healthcare access in order to improve vaccine uptake among migrant populations.

Our study revealed that the majority of migrants (96.6%) received information about the COVID-19 vaccine from family and friends, while 19% relied on television, radio, or newspapers. Only 8.9% reported obtaining vaccine information from NGOs, and a mere 4.5% indicated that they learned about the vaccine through the public health system. Migrants who expressed doubts about the credibility of their information sources had significantly higher odds of being vaccine hesitant, while those who did not encounter negative information had lower odds of hesitancy.

These findings align with other studies (31, 32). For example, a cross-sectional study conducted among 800 undocumented migrants in the United States, Switzerland, Italy, and France found that migrants primarily accessed COVID-19 vaccine information through traditional media, such as TV and radio, followed by social media (31). Similarly, Troiano and Nardi’s review highlighted that migrants often rely on these sources for vaccine-related information (32). Furthermore, 73% of participants in that study accessed information through their community networks, which was identified as a key predictor of vaccine demand. This supports our findings, as reliance on community networks, including family and friends, significantly shaped migrants’ attitudes toward vaccines.

Other research has also underscored the role of family networks in shaping vaccine beliefs. Studies have shown that migrants whose family members were vaccine hesitant were more likely to share similar doubts about vaccine efficacy (25). Additionally, barriers to receiving accurate public health messaging, such as language difficulties and distrust in healthcare systems, have been identified as factors limiting vaccine motivation among certain refugee and migrant groups (28, 29). In the local context of Trinidad and Tobago, a cross-sectional study on HIV testing among Venezuelan migrants found that reliance on family and friends was a strong predictor of health service uptake (33). This emphasizes the significant role of social networks in shaping health behaviors among migrants. These findings suggest that family and community networks can be both facilitators and barriers to vaccine uptake, depending on the type of information shared. Therefore, improving trust in public health messaging and ensuring that credible information reaches migrants and other vulnerable populations through trusted community channels is crucial to increasing vaccine acceptance.

### 4.1 Strengths and limitations

Random sampling from the AMMR database was used to reach a study population of Venezuelan migrants, a hard-to-reach population in Trinidad and Tobago. At the time of our study, the UNHCR estimated that 34,100 Venezuelan migrants and refugees were residing in Trinidad and Tobago. The database from which or sample was drawn contained information on 19,000 of these migrants (55.7%) across the country, thus making it a fair representation of this subpopulation. The study employed the use of bilingual nurses to overcome language, trust, and literacy barriers to participation and, therefore, contributed to the uptake of responses by the target population. While our study sample size was reached using telephone interviewing, some participants contact numbers were out of service which required revisiting the database of contacts to acquire additional information, and in some cases additional study participants. Survey items focused on barriers and facilitators to accepting the COVID-19 vaccine which may have introduced some bias in the migrants’ responses. Dichotomous items were primarily used for migrants’ perceptions and beliefs to allow for quick responses without ambiguity that can sometimes arise in multi-point Likert scales or more complex response options. However, given that migrants’ attitudes toward the COVID-19 vaccine were being measured, the dichotomous items may force participants into an oversimplified position; limiting our ability to capture strengths of beliefs.

## 5 Conclusion

Ensuring equitable access to vaccinations for refugees and migrants is critical to achieving broader vaccine coverage goals and reducing deaths from vaccine-preventable diseases. Globally, migrants and refugees face barriers in accessing healthcare, particularly immunization services, and these challenges are even greater for undocumented migrants who lack appropriate documentation. The COVID-19 pandemic exposed significant health inequities, with vulnerable populations such as migrants and refugees often left out of vaccination efforts. The COVID-19 pandemic unveiled several health inequities, including vaccination, underscoring the need for targeted strategies to reach under-immunized and marginalized groups of refugees and migrants. Interventions to increase vaccine uptake are only partially understood, highlighting the need for further research to improve coverage, reduce vaccine hesitancy and under-immunization. To reduce and address barriers to COVID-19 vaccine uptake among migrants, countries need clear vaccination policies that meet their needs. Healthcare professionals in the public health system will require training to better understand and respond to migrants’ health needs.

In Trinidad and Tobago, the rollout of our national pandemic response by the Government was not inclusive of a migrant specific vaccination plan. Strategies to increase update of vaccinations by migrants should be guided by policies with a focus on developing and rolling out vaccination literacy, targeting migrants. Given our findings, it is recommended that tailored messages about vaccination, community-based interventions and convenient local clinics can encourage underserved groups to have vaccinations. Greater consideration must also be given to the influence of social media-based communication as a source of information about vaccinations, mass communication campaigns through the public sector, humanitarian and NGO service providers. These combined efforts can reduce vaccine hesitancy and improve health outcomes for underserved migrant populations during future health crises.

## Data Availability

The raw data supporting the conclusions of this article will be made available by the authors, without undue reservation.
